# Energy and Protein Requirements of Growing Lambs in Colombian Highlands

**DOI:** 10.3390/ani14142117

**Published:** 2024-07-20

**Authors:** Yesid Avellaneda, Edgar Mancipe, Juan Vargas, Diego Manriquez

**Affiliations:** 1 Corporación Colombiana de Investigación Agropecuaria (AGROSAVIA), C.I. Tibaitatá, Km 14 Vía Mosquera, Mosquera 250047, Cundinamarca, Colombia; yavellaneda@agrosavia.co (Y.A.); emancipe@agrosavia.co (E.M.); 2 AgNext, Department of Animal Sciences, Colorado State University, Fort Collins, CO 80523, USA; juan.vargasmartinez@colostate.edu

**Keywords:** energy requirements, feed restriction, protein requirements, sheep production

## Abstract

**Simple Summary:**

Determination of nutrient requirements in sheep is essential to generate appropriate recommendations for dietary formulation, promoting sustainable systems. However, there is limited information regarding the energy and protein requirements of growing lambs in the Colombian highlands. Twenty-two growing lambs were enrolled in this experiment. Four lambs were slaughtered at the beginning of the experiment to determine the lambs’ energy and protein baseline concentration. The remaining lambs received different amounts of feed and were slaughtered to determine the energy and protein accumulation during the evaluation period. The maintenance energy was 82.3 kcal/kg BW^0.75^, while maintenance and gain energy efficiencies were 0.72 and 0.29, respectively. In addition, the crude protein requirement for maintenance was 1.78 g/kg BW^0.75^, and the nitrogen efficiency for maintenance and gain protein was 0.41 and 0.27, respectively. The maintenance requirement of energy was greater, while protein was similar to values reported in the literature; however, the efficiency of energy and protein use was lower in growing lambs. These results suggested that estimating energy and nitrogen requirements for growing lambs must be adjusted in Colombian highland conditions.

**Abstract:**

Determining the nutritional requirements of lambs is necessary to formulate balanced rations and contribute to sustainable sheep production systems. However, limited information is available regarding the nutritional requirements of growing lambs in the Colombian highlands. Twenty-two Romney Marsh males were used. Initially, four lambs were slaughtered at 20 kg body weight (BW). The remaining 18 lambs were distributed into two groups, according to BW (light: 20 BW and heavy: 30 kg BW). Lambs were randomly allocated into one of three different nutritional schemes, according to voluntary intake (no restriction, and 25 and 50% restriction). All the animals received a totally mixed ration. When a voluntary-fed lamb gained 10 kg BW, the two other lambs restricted to 25 and 50% were slaughtered and dissected into components to determine protein and gross energy concentration. Energy and protein accumulation were analyzed through regression analysis. The net energy for maintenance was 82.3 kcal/kg BW^0.75^, while the energy efficiencies for maintenance and gain were 0.72 and 0.29, respectively. Crude protein for maintenance was 1.78 g/kg BW^0.75^, and the nitrogen efficiencies for maintenance and gain were 0.41 and 0.27, respectively. The maintenance requirement of energy was greater, while protein was similarly related to values reported in the literature; however, the efficiency of energy and protein use was lower in growing lambs. These results suggested that estimating energy and nitrogen requirements for growing lambs must be adjusted in Colombian highland conditions.

## 1. Introduction

In Colombia, sheep production has increased due to the intensification of ovine systems, but productivity and profitability are aspects that require further improvement. There are around 1,449,705 sheep distributed mainly in the regions of La Guajira (45.3%), Boyacá (12.8%), Cesar (12.7%), and Magdalena (9.1%) [1]. In the Colombian highlands (i.e., above 2600 m above sea level), sheep production systems employ wool-type animals that produce both meat and wool under grazing conditions. However, these systems employ traditional practices with low-intensity levels and profitability [2]. Feeding is a critical component in the production costs of the production systems [3]. In this regard, efficient feed management is a strategy to increase animal performance and business profitability, decrease environmental impact, and improve food security [4,5]. Therefore, it is necessary to optimize the nutritional balance of sheep’s diets to ensure the sustainability of sheep production systems and improve the quality of life of sheep farmers in the Colombian highlands.

The Agricultural and Food Research Council [6], the Commonwealth Scientific and Industrial Research Organization [7], and the National Research Council [8] are well-recognized committees that define nutritional models for domestic animals, such as ovine. These models allow for the estimation of the dry matter intake and nutritional requirements of sheep. In addition, these models recognize that nutrient requirements and efficiencies vary according to the physiological state, sex, and breed of the ovine and the management and environmental conditions [7]. In this regard, the definition of the nutrient requirements of growing lambs requires the determination of the chemical composition of deposited tissues under local management and environmental conditions [9]. However, no published data regarding the nutritional requirements for lambs in the Colombian highlands exist. The lack of local information could affect the estimation of nutrient requirements and the diet formulation, resulting in poor animal performance and low economic efficiencies. Therefore, the aim of this study is to define the energy and protein requirements of growing lambs adapted to the Colombian highland regions. The experimental hypothesis was that the requirements and efficiencies of energy and protein of adapted-growing sheep in the highland regions of Colombia are different from previous studies due to the variation in the genetic potential of adapted breeds.

## 2. Materials and Methods

The Bioethics Committee of the Colombian Corporation of Agricultural Research (Agrosavia) approved the animal management and procedures employed in this experiment (045-2022).

### 2.1. Experimental Site and Treatments

The experiment was conducted in the Nutritional and Feeding Unit at the Tibaitatá Research Center (Agrosavia), located in the municipality of Mosquera, Colombia, at 2516 m above sea level. The mean temperature was 16 °C, and the relative humidity was 75%.

A total of 22 Romney Marsh growing intact male lambs were purchased from a local sheep production system. The 20 kg lambs were recently weaned, while the 30 kg lambs were weaned approximately 2 months early. Heavy lambs were grazing Kikuyu grass (*Cenchrus clandestinus*) and supplemented with a commercial concentrate (Italovinos, Italcol, Colombia). Previous to the experiment, all lambs were dewormed (Fenbendazol, Vecol, Colombia). Four lambs with 20 ± 0.5 kg of body weight (BW) were initially slaughtered to establish the reference concentration of protein and energy in the different tissues. The remaining lambs were distributed by BW into two different groups. Nine lambs with 20 ± 0.7 kg of BW were in the light group, while nine lambs with 30 ± 1.3 kg of BW were in the heavy group. Three lambs from each group were randomly allocated to one of the three different nutritional schemes according to voluntary intake: no restriction, 25% restriction, and 50% restriction. Lambs were allocated in individual concrete pens (approximately 3 m^2^) that were bedded with rice husk and had an automatic drinking source and container feeder.

### 2.2. Feeding Treatments and Animal Management

The diet was formulated to supply the maintenance requirement and 240 g/d of BW gain for a 25 kg lamb in the no-restriction feed intake treatment (half the initial weight of the two groups) [8]. The chemical composition of ingredients was evaluated at the Chemical Analytical Laboratory of Agrosavia. The Angleton grass (*Dichanthium aristatum*) hay was chopped (less than 2 cm) and mixed with other ingredients to obtain a total mixed ration (Table 1). The digestible energy of each feed resource was estimated as the product of the in vitro digestibility and 4.4 kcal/g [10]. Metabolizable energy was estimated as the digestible energy of each resource multiplied by either 0.81 for forages (corn silage and Angleton grass) or 0.85 for concentrate feed resources (Table 1; [7]). Intake of ME was calculated as the product between the ME value of the ration and the average dry matter consumption recorded during the evaluation period, which was scaled to the average metabolic body weight of each animal during the study.

The feed offer and the refusal of each feeder were measured daily to calculate the feed intake of the animals in the voluntary intake group, with which information the offer for the next day to the other individuals was restricted at 25 and 50%. The diet was supplied daily at 8:00 h. Once a voluntary-fed lamb achieved 10 kg of BW gain (i.e., light group: 30 kg of BW or heavy group: 40 kg of BW), the two other lambs restricted to 25 and 50% in the group were slaughtered. Before they were slaughtered, lambs did not receive food or drinking water for 18 h. Then, lambs were weighed on a digital scale (CPW-Plus 75, Adam brand, UK) to determine the final BW.

The lambs were slaughtered following the protocols of Title 7 of the Health Code for Terrestrial Animals of the World Organization for Animal Health [11]. The procedure consisted of stunning the lambs with a captive bolt pistol, causing a concussion. Subsequently, they were killed by exsanguination. The blood was weighed, and a sample was subsequently taken for conservation and analysis. The gastrointestinal tract was washed to remove the nutritional contents and weighed after the washing water was drained. The skin with wool was removed and subsequently, from different representative areas of the body, the wool was separated from the skin. Each animal was dissected into the following components: head and hooves, wool and skin, white viscera (rumen, reticulum, omasum, abomasum, and intestines), red viscera (heart, kidneys, trachea and lungs, vessel, liver and gallbladder, and spleen and pancreas), reproductive organs, mesenteric fat, and carcass. Each component was weighted, and representative samples were collected for grinding with a meat grinder (4 mm; P-22, Braher, Spain). Then, two subsamples of each component were homogenized and lyophilized for further analysis. The samples were sent to the Agrosavia analytical chemistry laboratory, where they were lyophilized and subsequently processed to determine fat, protein, ash, and gross energy in accordance with the AOAC procedures [12].

### 2.3. Energy and Protein Partition

The energy and protein partitions were estimated using the equations reported in the literature (Table 2; [9,13]). Heat production was calculated as the difference between the metabolizable energy intake and the retained energy, adjusted by the metabolic weight of each animal.

### 2.4. Statistical Analysis

Energy and protein partitions were analyzed through regression analysis using the REG procedure of SAS 9.4 [14]. Each animal was considered an experimental unit. Homoscedasticity, independence, and normality of the residuals were evaluated for each analysis by plotting residuals against predicted values.

## 3. Results

The results of the productive performance of the lambs according to initial BW and feeding schemes are presented in Table 3. As expected, the animals that were not restricted achieved a greater daily weight gain and final weight, but those that experienced the greatest restriction presented a better gain/feed intake ratio. Furthermore, feed efficiency was greater in animals in the lighter group.

**Figure 1:**
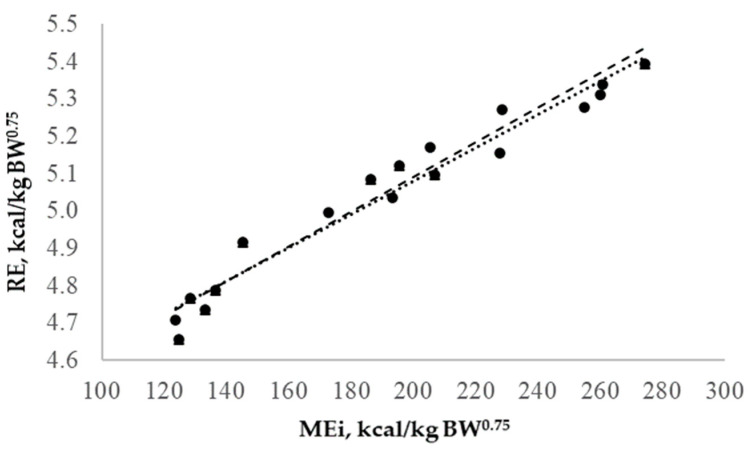
Relationship between metabolizable energy intake (MEi) and the natural logarithm of heat production (LnHP) of growing lambs. Light (●) and heavy (▴) growing lambs. Linear regressions for light (· · · ·) and heavy (- - -) groups.

Predictions of the EBW and EADG were described as the following equations: EBW = 0.080 (0.900) + 0.795 (0.027) × BW, R^2^: 0.594, and EADG = −3.59 (2.01) + 0.826 (0.014) × ADG, R^2^: 0.644. In addition, there was a linear relationship between the metabolized energy intake (MEi, kcal/kg EBW^0.75^) and the natural logarithm of the heat production (NLHP, Figure 1), and it was described as NLHP = 0.004 (0.0002) × MEi + 4.41 (0.061), (R^2^ = 0.92). In the analysis of homogeneity of slopes, it was found that the interaction between weight group and metabolizable energy consumption was not significant (*p* = 0.52), which confirms a single equation for all animals.

**Figure 2:**
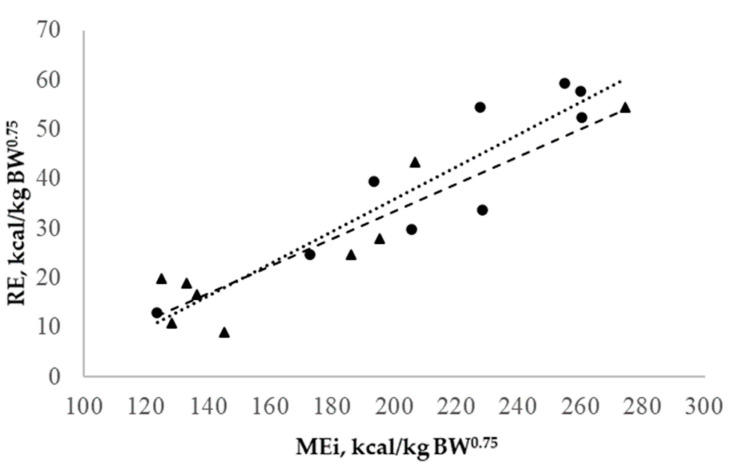
Relationships of metabolizable energy intake (MEi) and retained energy (RE) of growing lambs. Light (●) and heavy (▴) growing lambs. Linear regressions for light (· · · ·) and heavy (- - -) groups.

The estimation of the net energy for maintenance (NE_m_) was 82.3 kcal/kg BW^0.75^ or 97.6 kcal/kg EBW^0.75^. Also, the retained energy (RE) and the MEi showed a linear relationship (Figure 2), which was described as the following equation: RE = 0.29 (0.043) × MEi—33.73 (10.470), (R^2^ = 0.82). Like heat production, the slopes for energy retention were homogeneous (*p* = 0.50) for the heavy and light BW groups.

The metabolizable energy requirement for maintenance was 116.3 kcal/kg BW^0.75^ or 138.3 kcal/kg EBW^0.75^. Thus, the partial energy efficiency for maintenance (k_Em_) was 71.7%. The energy efficiency of gain (k_Eg_) was 29.4%. The solution to the requirement of net energy for gain (kcal/kg BW^0.75^/d) was REN_g_ = 10^−0.73 (0.56)^ × EADG^1.0976 (0.94)^ (R^2^ = 0.46) In this regard, the energy requirements to gain 100, 200, or 300 g/d of ADG were calculated as 181, 398, and 626 kcal/d for lambs of 20 kg, and 245, 538, and 847 kcal/d for lambs of 30 kg, respectively.

**Figure 3:**
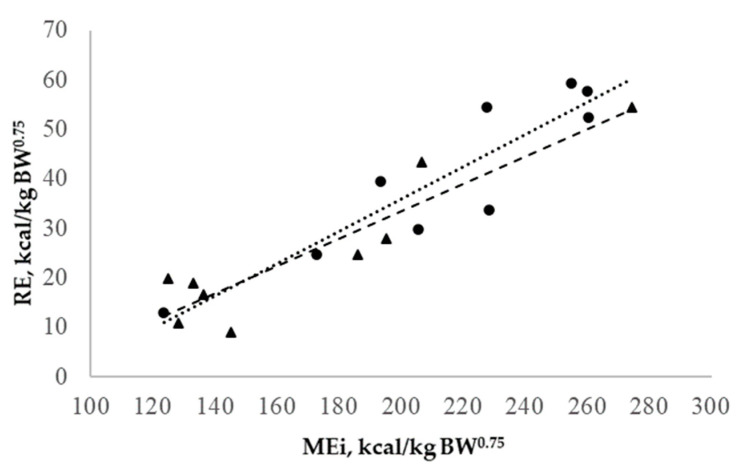
Relationships of the retained nitrogen (RN) and nitrogen intake (Ni) of growing lambs. Light (●) and heavy (▴) growing lambs. Linear regressions for light (· · · ·) and heavy (- - -) groups.

The retained nitrogen (RN) and the nitrogen intake (Ni) showed a linear relationship (Figure 3), which was statistically similar between the heavy and light BW groups. This relationship was described in the following equation: RN = 0.331 (0.033) × Ni—285.9 (65.4), (R^2^ = 0.85). In this case, the nitrogen requirement for maintenance (Nm) was 285.9 mg/kg BW^0.75^ or 1.78 g CP/kg BW^0.75^, and the nitrogen retention efficiency was 33.1%.

**Figure 4:**
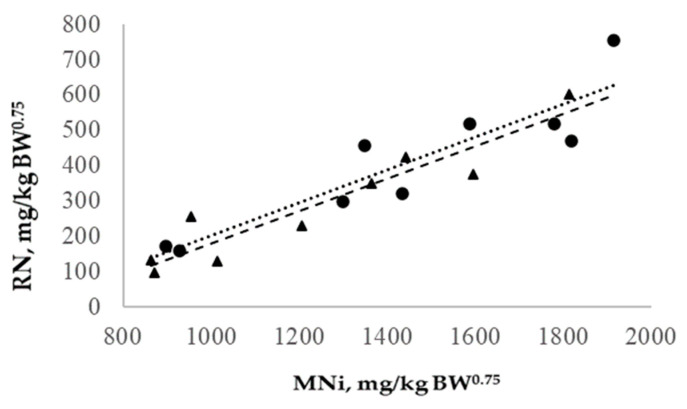
Relationships of the retained nitrogen (RN) and metabolizable nitrogen intake (MNi) of growing lambs. Light (●) and heavy (▴) growing lambs. Linear regressions for light (· · · ·) and heavy (- - -) groups.

The retained nitrogen and metabolizable nitrogen intake (MNi) showed a linear relationship (Figure 4) described in the following equation: RN = 0.461 (0.039) × MNi—321.9 (65.4), (R^2^ = 0.83). In this regard, the metabolizable nitrogen for maintenance was 698.2 mg/kg BW^0.75^, while the nitrogen maintenance efficiency was 40.9%. Lastly, the relationship between the RN, EBWG, and RE was described with the following equation: RN (mg N/kg BW^0.75^) = 144.5 (70.4) + 1.323 (0.358) × EADG + 1.259 (0.654) RE, R^2^ = 0.68. Therefore, the nitrogen requirements for a 20 kg lamb with 100, 200, or 300 g/d of ADG were 2623, 3975, and 5,44, and for a 30 kg lamb were 3555, 5388, and 7244 mg N/d, respectively.

## 4. Discussion

The response to energy and nitrogen use was statistically similar between the two bodyweight categories evaluated in this study, indicating that in the evaluated weight range (i.e., 20 to 40 kg), the requirements are similar per unit of metabolic weight. On the other hand, the net energy for maintenance accounts for the energy to support basal metabolism and heat production to maintain vital metabolic functions [15,16]. In this experiment, the net energy for maintenance was 82.3 kcal/kgBW^0.75^, which is higher than values that have been reported for crossbred sheep (54.5 kcal/kg BW^0.75^ [17]), Mora nova breed (52.4 kcal/kg BW^0.75^ [18]), meat-type lambs (52.4 kcal/kg BW^0.75^ [19]), and the NRC model (56 kcal/kgBW^0.75^ [8]). Also, it is higher than other studies that report maintenance values of 66 kcal/kgBW^0.75^ [7] and 68.5 kcal/kgBW^0.75^ [20]. Although there are no studies evaluating the energy requirements of growing lambs in the Colombian highlands, the high energy requirement could be associated with the altitude and cold conditions where this experiment was conducted. Although sheep have developed different mechanisms to adapt to different altitudes [21], it is recognized that this environmental factor affects animal performance [22]. In this regard, exposure to hypoxic conditions and low temperatures results in lower reproductive performance and weight gain in lambs [23,24,25]. In addition, the metabolizable energy for maintenance was 116.3 kcal/kgBW^0.75^, which is higher than the values reported for Baluchi lambs (81.7 kcal/ kg BW^0.75^ [26]) and crossbred lambs (82.3 kcal/kg BW^0.75^ [27]), but similar to British breed lambs (109.9 kcal/kg BW^0.75^ [28]). In this experiment, the partial energy efficiency for maintenance was 0.72, greater than 0.67 [17,27] or 0.64 [8] but similar to 0.70 [6,29].

The energy requirement for gain is associated with the physiological condition of the lambs. For example, the energy requirements for non-castrated and castrated male and female lambs with 20 kg of BW and 100 g of daily body gain were 191, 198, and 276 kcal/kg BW^0.75^, respectively [9]. Females have more energy requirements due to a higher fat deposition [30]. In the current study, the estimated requirement for a male lamb with 20 kg of body weight and 100 g of daily weight gain was 181 kcal/kg BW^0.75^, similar to the 191 kcal/kg BW^0.75^ [9] but higher than the 122 kcal/kg BW^0.75^ [8]. The gain efficiency in this study was 0.29, and it was superior to 0.21 [18]. However, several studies report that the efficiency for energy gain varies between 0.36 and 0.58 [6,17,28,31,32], higher than the value observed in the current study. Variations in energy efficiency could be explained due to differences in the energetic concentration of the diet, nutrient digestion and absorption, and composition of the body gain [33]. In addition, further work should consider the mature BW to correct the estimation of the energy requirements for gain.

In the current work, the net protein requirement was 286 mg/kgBW^0.75^, similar to the 277, 293, and 303 mg/kgBW^0.75^ reported in Brazilian ovine systems [18,34,35]. Galvani et al. [31] reported that the net protein requirement was 287 g/kg BW^0.75^, and it was not affected by the dietary characteristics. The metabolizable nitrogen efficiency for maintenance was 0.41, lower than 0.67 [8] and 1 [6] but similar to 0.41 [35]. Nevertheless, Galvani et al. [31] reported that nitrogen efficiency varies between 0.45 and 0.56, and it is affected by the quality of the diet. Also, the nitrogen efficiency for weight gain in this work was 0.27, similar to 0.28 [21], but lower than 0.59 [6], 0.7 [7,8], or 0.75 [22]. Methodological aspects may explain differences in nitrogen efficiency. In this regard, restricted animal diets may limit animal growth potential due to energetic limitations [36] associated with changes in rumen microbial populations, fermentation products, and the microstructure of the gastrointestinal tract [37,38]. The maximum efficiency has been suggested to be achieved when the protein is the first limiting nutrient, and not the energy [7]. For this reason, it is necessary to incorporate the evaluation of different diet compositions, rumen fermentation dynamics, and protein fractions to improve the nutrient efficiency evaluation.

## 5. Conclusions

In conclusion, net energy for maintenance (82.3 kcal/kg BW^0.75^) was higher than previous reports and may be associated with the altitude conditions of this study, while energy efficiency for maintenance (0.72) and nitrogen requirement (285.9 mg/kg BW^0.75^) were similar to the previous results reported in the literature. However, the energy efficiency for gain (0.29) and the protein efficiencies for maintenance and weight gain (0.41 and 0.27, respectively) were lower than the recommendations in the literature. These results suggest that the energetic models may predict the energy efficiency and the nitrogen requirements for maintenance, but the current models may not explain the energy requirements for maintenance and the efficiency of nitrogen for maintenance and gain. Thus, further research is needed to properly determine the energy and nitrogen requirements of other physiological conditions (e.g., pregnancy or lactation) in sheep in the Colombian highlands.

## Figures and Tables

**Table 1:** Inclusion, in vitro dry matter digestibility (IVDMD), and chemical composition of the totally mixed ration fed to growing lambs.

Inclusion	% of DM Basis	IVDMD, %
Corn silage	73.3	64.9
Angleton grass hay	9.8	54.2
Soybean meal	8.7	94.3
Wheat middlings	4.7	84.8
Soybean (extruded)	2.1	92.8
Fish meal	0.9	77.6
Limestone	0.1	-
Salt	0.3	-
**Calculated composition** **^1^**	**On DM basis**	
ME, Mcal/kg	2.4	
NEm, Mcal/kg	1.4	
NEg, Mcal/kg	0.8	
CP, %	17.4	
RDP, %	11.3	
MP, %	10.9	
NDFe, %	27.9	
TDN, %	72.8	
Ca, %	0.54	
P, %	0.47	
**Determined composition**	**On DM basis**	
GE, Mcal/kg	4.5	
CP, %	15.2	
NDF, %	38.4	
Ca, %	0.49	
P, %	0.45	

^1^ ME: metabolizable energy; NEm: net energy for maintenance; NEg: net energy for gain; CP: crude protein; RDP: rumen degradable protein; MP: metabolizable protein; NDFe: effective neutral detergent fiber; TDN: total digestible nutrients; GE: gross energy; NDF: neutral detergent fiber.

**Table 2:** Defined equations for determining energy and protein partitions of growing lambs.

Variable	Equation ^1^	Notes
Empty body weight (EBW, kg)	a + b × Body weight (BW, kg)	
Empty average diary gain (EADG, g/d)	a + b × Average daily gain (ADG, g/d)	
Ln (heat production (kcal/kg BW^0.75^/d))	a + b × Metabolizable energy intake (ME_i_, kcal/kg ^BW0.75^)	The intercept of the antilogarithm regression was used to estimate the requirement of the net energy for maintenance (NE_m_)
Retained energy (RE, kcal/kg BW^0.75^)	a + b × Metabolized energy intake (ME_i_; kcal/kg BW^0.75^)	The metabolizable energy for maintenance (ME_m_) was calculated as the regression solution without energy retention. The energetic efficiency for maintenance (k_Em_) was calculated using the NE_m_/ME_m_ ratio. Finally, the regression slope was considered the energy efficiency of gain (k_Eg_).
Requirement of net energy for gain (NE_g_, kcal/kg BW^0.75^/d)	10^a^ × EADG^b^ (kg)	
Retained nitrogen for weight gain (mg/kg BW^0.75^)	a + b × Nitrogen intake (mg/kg BW^0.75^)	The intercept corresponds to endogenous and metabolic nitrogen losses (N_m_). The slope was considered the efficiency of using protein above maintenance (k_Ng_)
Retained nitrogen for maintenance (mg/kg BW^0.75^)	a + b × Nitrogen for metabolizable protein (mg/kg BW^0.75^)	The metabolizable protein for maintenance (MN_m_) was calculated as the regression solution without protein retention. The N_m_/ M_Nm_ ratio was the maintenance efficiency (k_Nm_)
Requirement of nitrogen for gain (mg/kg BW^0.75^)	a + b × EBWG + c × RE	

^1^. a,b,c: Regression coefficients.

**Table 3:** Growth performance of growing lambs under different levels of feed restriction.

Item	Feed Restriction, %			Live Weigh Class, kg	
	0	25	50	20–30	30–40
Lambs	6	6	6	9	9
Live weight, kg					
Initial	24.6	24.3	24.0	19.3	29.3
Final	39.2	35.9	33.7	31.8	40.7
Weight gain, kg/d	0.16	0.14	0.12	0.15	0.13
DM intake, kg	1.38	1.02	0.71	0.95	1.12
Gain to feed ratio, kg/kg	0.12	0.12	0.17	0.16	0.12

## Data Availability

The data that are presented in this paper are available on request to the corresponding author.
